# A Rare Presentation of Native Aortic Valve Endocarditis Secondary to Streptococcus gordonii: A Case Report

**DOI:** 10.7759/cureus.111541

**Published:** 2026-06-26

**Authors:** Franciela Golden, Keerthana Reddy Pagidela, Nadine Elhage, Trent Davis, Kelley Devoe, Aleksandra Murawska Baptista, Abhinav Singla

**Affiliations:** 1 Department of Family Medicine, Mayo Clinic, Jacksonville, USA; 2 Department of Internal Medicine, Poznan University of Medical Sciences, Poznan, POL; 3 Department of Hospital Internal Medicine, Mayo Clinic, Jacksonville, USA

**Keywords:** bacteremia, case report, infective endocarditis, native aortic valve, septic emboli, spinal abscess, streptococcus gordonii

## Abstract

*Streptococcus gordonii* is an alpha-hemolytic Gram-positive bacterium most commonly present in the oral cavity and gastrointestinal tract. Despite an increase in the incidence of infective endocarditis, *S. gordonii* cases reported on a native aortic valve are relatively uncommon. Reported cases have been linked to spondylodiskitis, intra-abdominal infection, and oral procedures. A 79-year-old male was admitted for acute kidney injury, diarrhea, and dehydration, and was found to have *S. gordonii* bacteremia with endocarditis. A 1.7 cm × 0.9 cm vegetation on the native aortic valve was revealed, and subsequent multiple septic emboli on brain magnetic resonance imaging (MRI) were discovered after an incident of acute mental status change. The patient was successfully treated with intravenous ceftriaxone and later required aortic valve replacement. A possible source of the bacteremia was a trigger point injection to the lumbar spine, which was consistent with MRI lumbar spine findings of a spinal abscess. However, other possible sources include a dental procedure, cholecystitis with concern for cholelithiasis, and colonoscopy with polypectomy, both incidences within eight weeks of the hospitalization. This case illustrates the importance of thorough history taking and consideration for an echocardiogram to rule out endocarditis if *S. gordonii* bacteremia is present, in addition to close monitoring and outpatient follow-up post-diagnosis. Considering an increasing number of procedures involving the oral cavity, spine, and abdominal area, we hope that this case will bring awareness to the diagnosis of *S. gordonii* endocarditis.

## Introduction

*Streptococcus gordonii* is an alpha-hemolytic Gram-positive bacterium most commonly present in the oral cavity and gastrointestinal tract [1]. It is a member of the viridans group streptococci, a low-virulence group of the oral mucosa, but well recognized as a causative agent of infective endocarditis, especially when access to the bloodstream is gained, particularly after dental or mucosal procedures. It relies on direct and indirect binding of fibrinogen and fibronectin using serine-rich glycoprotein adhesives as a virulence factor. This facilitates the formation of vegetations, especially seen in the setting of fibrinogen-rich areas present on cardiac valves [2]. Native valve endocarditis refers to infection of the endocardial surface of a heart valve that has not been surgically replaced, most commonly resulting from bacteremia seeding a valve already altered by turbulent flow, degenerative changes, or pre-existing structural abnormalities.

Despite an increase in the incidence of infective endocarditis, there is a limited number of *S. gordonii* endocarditis cases reported on a native aortic valve. A 2024 case series and literature review of 25 reported cases of *S. gordonii* endocarditis found that the mitral valve was the most commonly affected site (52% of cases), with isolated aortic valve involvement and combined aortic-mitral involvement together accounting for the remainder of cases; septic emboli were identified in 36% of cases, most often to the brain (20%) or spine (16%), with the latter causing discitis [3]. This indicates that, while *S. gordonii* endocarditis is uncommon overall, isolated native aortic valve involvement, as seen in our patient, represents a less frequent subset of an already rare disease, which may contribute to underrecognition and delayed diagnosis.

Previous cases of *S. gordonii* have been associated with spondylodiskitis, intra-abdominal infection, and oral procedures [4]. We present a case of *S. gordonii* endocarditis with aortic valve vegetation on a native aortic valve, complicated by septic embolism. This case is clinically important because it demonstrates how *S. gordonii* endocarditis can arise from any of the several distinct invasive procedures occurring close together in time, complicating efforts to identify a single source, and because it illustrates the value of prompt echocardiographic evaluation once *S. gordonii* bacteremia is identified, even in the absence of an audible murmur. This case is particularly unique because the patient had multiple possible sources for the bacteremia, including a colonoscopy with polypectomy, an oral procedure eight weeks before presentation, and a spinal trigger point injection four weeks before presentation, resulting in spinal abscess formation. This case was prepared following the CARE guidelines (https://www.care-statement.org).

This article was previously presented as a poster presentation in the 6th edition of the World Congress of Infectious Diseases on June 25, 2024.

## Case presentation

A 79-year-old male presented to the emergency department of the Mayo Clinic, Jacksonville, with the chief complaint of diarrhea, nausea, and poor oral intake. His past medical history was significant for erosive osteoarthritis (L4-S1), diffuse lumbar spondylosis with severe foraminal stenosis (L4-S1), atrial fibrillation, coronary artery disease with a coronary artery bypass graft (CABG) history, and type 2 diabetes. In the Emergency Department, the physical examination was significant for tachycardia with irregular rhythm, increased bowel sounds, pain on palpation to the L2-S1 area, and decreased deep tendon reflexes in the lower extremity. Vital signs included blood pressure of 93/58 mmHg and heart rate of 118 beats/minute. Initial laboratory evaluation demonstrated abnormalities in renal function, electrolyte balance, and hepatic enzymes (Table 1). Computed tomography (CT) of the spine was performed, and it was unchanged from baseline. He was ultimately admitted to the inpatient service for acute kidney injury, diarrhea, and dehydration. Empirically, he was treated with cefepime and metronidazole for acute diarrhea with concerns for intra-abdominal processes.

**Table 1:** Initial laboratory findings on presentation.

Laboratory test	Result	Reference range
Hemoglobin	12.8 g/dL	14–18 g/dL
Creatinine	2.33 mg/dL	0.6–1.2 mg/dL
Blood urea nitrogen	71 mg/dL	8–23 mg/dL
Potassium	6.3 mEq/L	3.5–5.0 mEq/L
Aspartate aminotransferase	52 U/L	10–30 U/L
Alkaline phosphatase	252 U/L	30–120 U/L

On day one of hospitalization, the patient had right thoracic and lumbar back pain around the right upper quadrant (RUQ) radiating to the thoracic and lumbar spine. RUQ ultrasound showed acute cholecystitis with concerns for cholelithiasis, followed by a CT scan indicating a gallbladder diverticulum with acute inflammation. General surgery was consulted and recommended conservative treatment with IV ceftriaxone and metronidazole. The AKI resolved after IV fluids. Gastrointestinal stool panel was positive for *Clostridioides difficile*, and he was started on oral vancomycin 125 mg four times a day for 10 days.

On the second day of hospitalization, two out of two blood cultures grew *S. gordonii* at 14 hours. An early auscultation revealed no murmur until later. A concern for endocarditis warranted a transthoracic echocardiogram (TTE) and was significant for native trileaflet aortic valve endocarditis with a large mobile mass attached to the regurgitant surface of the aortic valve (right coronary cusp) protruding into the left ventricular outflow tract. Later in the day, he experienced a severe change in mental status. Rapid Response was called, and the patient was evaluated by Neurology. CT and CT angiography of the head were performed as part of the stroke protocol and did not show an acute intracranial finding. Due to the ongoing altered mental status, a brain magnetic resonance imaging (MRI) was performed that showed multiple subacute infarcts with concern for septic emboli (Figure 1). Subsequently, a transesophageal echocardiogram (TEE) was ordered for further workup. The TEE confirmed the vegetation findings on the TTE. The TEE showed a trileaflet aortic valve with mild regurgitation, measuring 1.7 cm × 0.9 cm (Figure 2).

**Figure 1:**
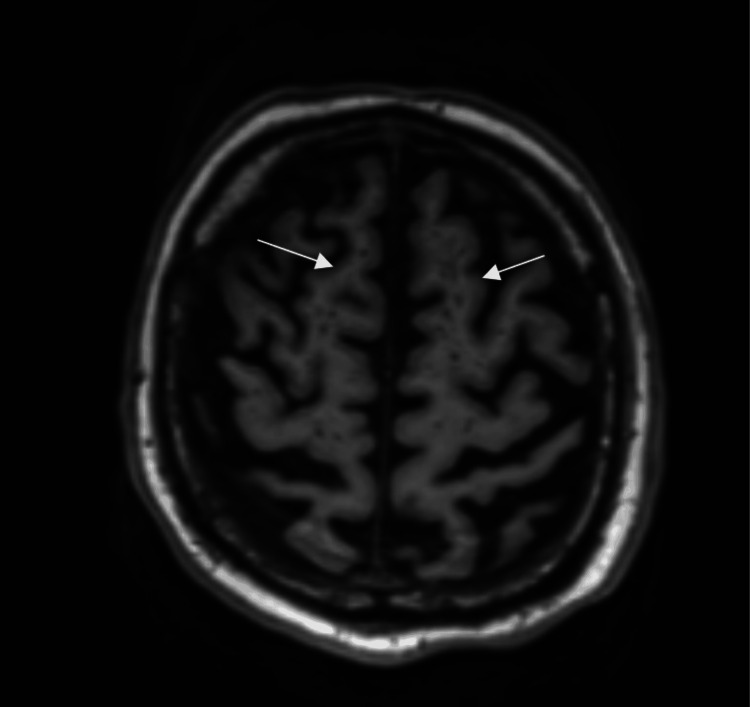
Brain MRI showing multiple subacute infarcts in the gyri. The arrow is pointing to multiple subacute infarcts in the gyri. This image was obtained due to the onset of an altered mental status.

**Figure 2:**
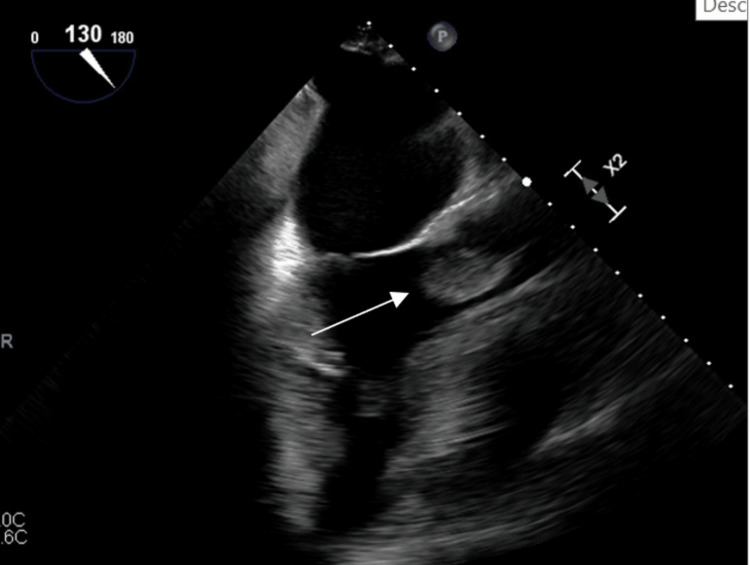
Mass on the aortic valve. Transesophageal echocardiogram with an arrow pointing at a mobile 1.7 cm × 0.9 cm mass on the aortic valve, confirming vegetation.

After discussing the findings with the patient, we found that the possible source for the bacteremia could have been related to a dental procedure or colonoscopy with polypectomy two months before bacteremia, or a trigger point injection four weeks prior. MRI of the lumbar spine showed septic left L4-S1 facet joints, likely with less severe involvement of the right L4-L5 facet joint, and possible early involvement of the right L5-S1 facet joint with a 1.8 cm × 0.5 cm abscess within the left paraspinal musculature at the L5 level, which correlated with the area of the trigger point injection (Figures 3, 4). A consultation with interventional radiology revealed that the abscess was too small to be drained, and Infectious Disease recommended IV ceftriaxone. Blood cultures grew Gram-positive cocci in chains with *S. gordonii* identified by FilmArray and confirmed with culture using matrix-assisted laser desorption/ionization time-of-flight mass spectrometry, while susceptibility testing showed the organism was sensitive to ceftriaxone with a minimal inhibitory concentration (MIC) of <0.25 µg/mL, penicillin with an MIC of <0.03 µg/mL, and vancomycin with an MIC of 1 µg/mL. As a result, IV ceftriaxone was continued as blood culture susceptibilities confirmed that SG was sensitive to the ceftriaxone. The patient’s mentation returned to normal within one to two days with no intervention besides ceftriaxone and continued Eliquis per Neurology recommendation. No antiseizure drugs were utilized. An MRI of the spine was repeated two months later, which revealed resolution of the abscess. Cardiothoracic surgery was consulted, given the patient’s native aortic valve endocarditis in the setting of prior CABG. The patient did not meet the criteria for surgical intervention, as he had no heart failure and had not failed medical therapy. Although his large, mobile vegetation was noted as a relative indication for intervention, the embolic risk was aimed to decrease significantly with continued therapy. Given his age, comorbidities, and frailty, he was not considered a candidate for cardiac surgery at that time. The preferred strategy was continued response to antibiotic therapy. He was discharged after 11 days in the hospital to an acute rehab facility for six weeks of IV ceftriaxone with follow-up appointments for repeat TTE and MRI of the lumbar spine, which revealed resolution of the abscess. A few months later, he was hospitalized for new-onset heart failure and was scheduled for a replacement of the aortic valve.

**Figure 3:**
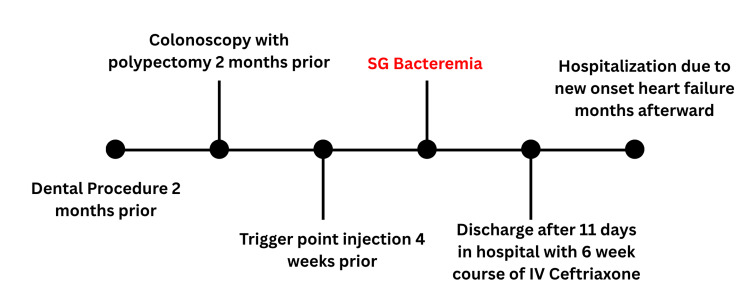
Timeline of procedures and hospitalization in a patient with Streptococcus gordonii endocarditis.

**Figure 4:**
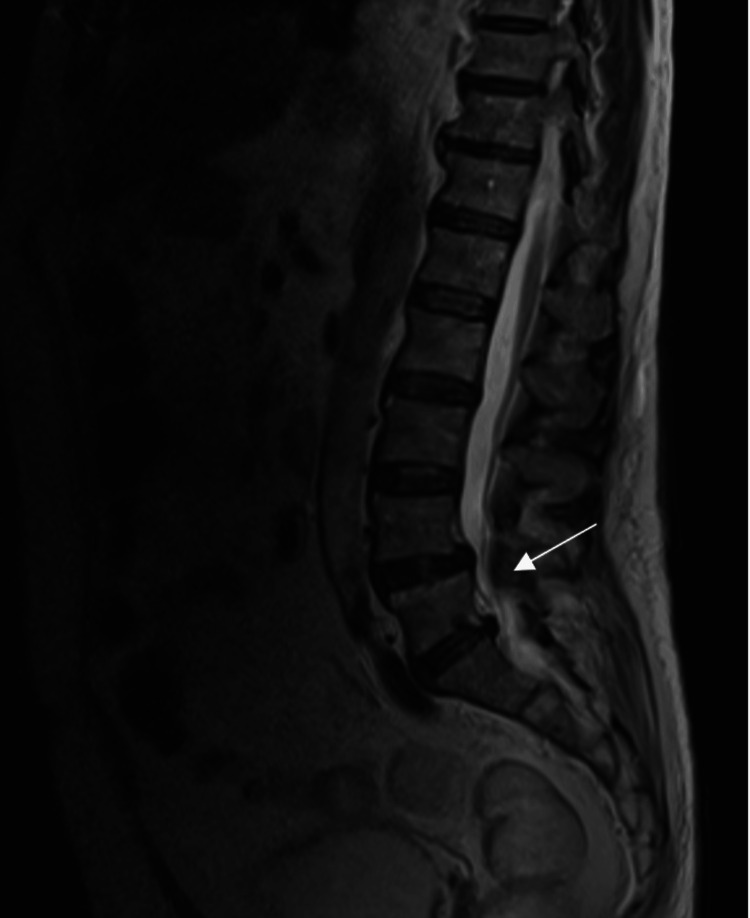
Abscess in the lumbar region. Lumbar MRI showing small an abscess measuring 1.8 cm × 0.5 cm at L5, with the arrow pointing to the abscess.

## Discussion

Native valve infective endocarditis has an evolving epidemiology over the years. In the United States, the incidence of infective endocarditis increased from 2000 to 2011 from 11 to 15 cases per 100,000 [4]. This rise in incidence is likely due to higher numbers of patients at risk for this disease. This includes older, diabetic, or hemodialysis patients. Additionally, advancements in medical interventions in aging populations have contributed to an altered epidemiological landscape, such as higher numbers of invasive procedures, possibly contributing to infective endocarditis.

Most common cases of endocarditis are due to *Staphylococcus aureus* and *Streptococcus* species [5]. *Staphylococcus aureus* is found to be prevalent in 40% of native valve infective endocarditis, with streptococci being 30-40%. *Streptococcus viridans* accounts for half of that percentage, while other streptococci species account for about 15% [6]. *S. gordonii* bacteremia is of a lower prevalence than *Streptococcus* bacteremia, but when present, it is associated with a higher likelihood of infective endocarditis [7]. This may be due to serine-rich glycoproteins that bind to human platelets and cardiac valves. It is important to recognize early due to the potential for a subacute, insidious clinical course, as the diagnosis may be missed in the initial stages [8]. Echocardiograms in *S. gordonii* bacteremia were scarcely utilized [9]. This may be due to criteria and guidelines highlighting other *Streptococcus* strains being more likely to cause endocarditis [10].

A limited number of cases addressing *S. gordonii* endocarditis have been identified [9]. To our knowledge, it includes a 31-year-old male who presented with sepsis preceded by a dental procedure, found to be caused by *S. gordonii* endocarditis with a mitral valve vegetation [11]. A case review included 15 cases of *S. gordonii* endocarditis between 1998 and 2017, with only five associated with aortic valve vegetation, unspecified whether native or prosthetic valves [12]. Recently, a case of *S. gordonii* endocarditis and aortic valve vegetation in a native aortic valve was reported in a young, immunocompromised male associated with COVID-19 infection [13]. Another patient was diagnosed with *S. gordonii* endocarditis after a cerebrovascular incident [14]. The mitral valve is identified to be the most commonly involved site in *S. gordonii* endocarditis, with isolated aortic valve involvement representing a smaller proportion of cases. *S. gordonii* is rarely reported but is associated with incredible morbidity [3].

Our case was unique in the sense that the patient had three possible sources of infection: colonoscopy with polypectomy, dental procedure, and lumbar trigger point injection within the eight weeks before the hospitalization. Additionally, a large 1.7 cm × 0.9 cm vegetation was present in a native aortic valve complicated by multiple septic emboli. A likely source for the *S. gordonii* endocarditis was the trigger point injection, as MRI of the lumbar spine identified a 1.8 cm × 0.5 cm abscess at L5, which correlated with the area of the trigger point injection. Without a direct culture from the abscess, the precise source of infection cannot be established. While it is difficult to accurately determine the cause of the infection, the key consideration is the complex and highly atypical clinical presentation of *S. gordonii*, particularly its association with multiple potential infection sources, such as in this patient. Echocardiographic evaluation can strengthen the diagnosis, as the presence of vegetations indicates further evaluation. Several plausible etiologic factors highlight the diagnostic difficulty in establishing the origin of infective endocarditis in such cases.

Treatment for endocarditis is largely tailored to isolated organisms from the blood culture. Our patient received multiple antibiotics during his hospitalization to account for *C. difficile* infection, acute cholecystitis, and *S. gordonii* endocarditis. The treatment for endocarditis included six weeks of IV ceftriaxone with close monitoring by the Infectious Diseases department. Three months later, the patient was scheduled for aortic valve replacement due to worsening cardiac function.

## Conclusions

*S. gordonii* bacteremia is not routinely screened for endocarditis. *S. gordonii* endocarditis in this case contributed to a multi-organ disease, including acute brain infarcts caused by septic emboli, aortic valve vegetation requiring future aortic valve replacement, and an extended hospitalization with rehabilitation discharge. At the time of *S. gordonii* bacteremia, there should be a consideration for an echocardiogram to rule out endocarditis, especially after multiple procedures in a short period of time. Appropriate history taking can lead to a diagnosis. Close monitoring and follow-up visits are crucial for patients with *S. gordonii* endocarditis. We hope that this case will bring awareness when seeing patients after multiple recent procedures.

## References

[1] Abranches J, Zeng L, Kajfasz JK (2018). Biology of oral streptococci. Microbiol Spectr.

[2] Fitzgerald JR, Foster TJ, Cox D (2006). The interaction of bacterial pathogens with platelets. Nat Rev Microbiol.

[3] Ali GA, Pérez-López A, Tsui C (2024). Streptococcus gordonii-associated infective endocarditis: case series, literature review, and genetic study. Clin Case Rep.

[4] Pant S, Patel NJ, Deshmukh A (2015). Trends in infective endocarditis incidence, microbiology, and valve replacement in the United States from 2000 to 2011. J Am Coll Cardiol.

[5] Pierce D, Calkins BC, Thornton K (2012). Infectious endocarditis: diagnosis and treatment. Am Fam Physician.

[6] Cahill TJ, Prendergast BD (2016). Infective endocarditis. Lancet.

[7] Baddour LM, Wilson WR, Bayer AS (2015). Infective endocarditis in adults: diagnosis, antimicrobial therapy, and management of complications: a scientific statement for healthcare professionals from the American Heart Association. Circulation.

[8] Bhatti A, Sagar V, McFaul K (2023). Streptococcus gordonii infective endocarditis complicated by brain abscess in a patient with a congenital bicuspid aortic valve: a case report. Eur Heart J Case Rep.

[9] Mun SJ, Kim SH, Huh K, Cho SY, Kang CI, Chung DR, Peck KR (2021). Role of echocardiography in uncomplicated Staphylococcus aureus catheter-related bloodstream infections. Medicine (Baltimore).

[10] Rhabneh L, Cohen T, Moosvi A, Ashour AM, Dwivedi S (2025). A rare presentation of infective endocarditis caused by Streptococcus gordonii following transcatheter aortic valve replacement. Cureus.

[11] Mosailova N, Truong J, Dietrich T, Ashurst J (2019). Streptococcus gordonii: a rare cause of infective endocarditis. Case Rep Infect Dis.

[12] Quan TP, Muller-Pebody B, Fawcett N (2020). Investigation of the impact of the NICE guidelines regarding antibiotic prophylaxis during invasive dental procedures on the incidence of infective endocarditis in England: an electronic health records study. BMC Med.

[13] Arbune M, Gurau G, Niculet E (2021). Prevalence of antibiotic resistance of ESKAPE pathogens over five years in an infectious diseases hospital from South-East of Romania. Infect Drug Resist.

[14] Chang CY, Gan YL, Radhakrishnan AP, Ong EL (2022). Acute abdomen revealed Streptococcus gordonii infective endocarditis with systemic embolism. Oxf Med Case Reports.

